# Exploring Concordance of Patient-Reported Information on PatientsLikeMe and Medical Claims Data at the Patient Level

**DOI:** 10.2196/jmir.5130

**Published:** 2016-05-12

**Authors:** Gabriel S Eichler, Elisenda Cochin, Jian Han, Sylvia Hu, Timothy E Vaughan, Paul Wicks, Charles Barr, Jenny Devenport

**Affiliations:** ^1^ PatientsLikeMe Cambridge, MA United States; ^2^ Genentech Medical Affairs South San Francisco, CA United States

**Keywords:** feasibility of data linking, patient-powered research network

## Abstract

**Background:**

With the emergence of data generated by patient-powered research networks, it is informative to characterize their correspondence with health care system-generated data.

**Objectives:**

This study explored the linking of 2 disparate sources of real-world data: patient-reported data from a patient-powered research network (PatientsLikeMe) and insurance claims.

**Methods:**

Active patients within the PatientsLikeMe community, residing in the United States, aged 18 years or older, with a self-reported diagnosis of multiple sclerosis or Parkinson’s disease (PD) were invited to participate during a 2-week period in December 2014. Patient-reported data were anonymously matched and compared to IMS Health medical and pharmacy claims data with dates of service between December 2009 and December 2014. Patient-level match (identity), diagnosis, and usage of disease-modifying therapies (DMTs) were compared between data sources.

**Results:**

Among 603 consenting patients, 94% had at least 1 record in the IMS Health dataset; of these, there was 93% agreement rate for multiple sclerosis diagnosis. Concordance on the use of any treatment was 59%, and agreement on reports of specific treatment usage (within an imputed 5-year period) ranged from 73.5% to 100%.

**Conclusions:**

It is possible to match patient identities between the 2 data sources, and the high concordance at multiple levels suggests that the matching process was accurate. Likewise, the high degree of concordance suggests that these patients were able to accurately self-report their diagnosis and, to a lesser degree, their treatment usage. Further studies of linked data types are warranted to evaluate the use of enriched datasets to generate novel insights.

## Introduction

Health researchers today have the luxury of choosing from a multitude of “big data” sources. Given the growing diversity of data, new insights and transformative potential arise when complementary sources can be linked at the individual level (with appropriate consents and protections) to provide a more holistic view of a patient’s journey with illness or to inform medical decisions [1].

Although the potential advantages of data linkage and integration of existing sources are tantalizing (eg, rapid, economical access), rigorous scrutiny is required to assure the resultant data, and analyses provide meaningful and valid insights. Key questions and potential hurdles must be addressed before data integration can be performed reliably at scale. Comparisons between data from clinical encounters (captured in electronic medical records, claims, or administrative records) and patient-reported data (captured via telephone interviews, in person interviews [2], mailed surveys, and in-clinic surveys [3,4]) have been undertaken in previous research. It has been recognized that there is a need to confirm data reported by patients engaged through Web-based methods. Cascade et al used manual medical chart review to validate data from 50 patients with gout registered on MediGuard.org and were able to verify diagnosis in 76% of patients who consented to participate [5]. However, paper record extraction was a barrier to validation with physicians’ offices being slow to return charts or, in some cases, refusing to take part altogether. The concordance of patient-reported data collected by an Internet-based registry with data captured from clinical care has not been well characterized in the literature.

In this paper, we describe the evaluation of the potential benefits and challenges of linking data from an online patient community (PatientsLikeMe [PLM]) with administrative claims data. Electronic medical and pharmacy claims data are collected as a result of clinical care, primarily for billing and reimbursement purposes. In addition, they are widely used by a variety of stakeholders for other purposes, including practice management, identifying market trends, studying medication compliance, conducting outcomes research, and building health economic models. However, such secondary uses of billing data may result in limitations when used for research purposes. For example, it may be difficult to infer causality because transactional data report *what* happened without *why* it happened. Furthermore, changes in billing data may reflect administrative errors or limitations in classifications rather than actual changes to patient condition, evaluations completed, and treatments given.

PLM data have been used in over 65 peer-reviewed scientific publications, particularly in the areas of patient-centered outcomes research [6], development of new patient-reported outcomes [7], and clinical trials [8]. As a patient-powered research network (PPRN) covering over 2000 diseases, the system emphasizes patient-reported data submitted in the Web and allows patients to enter detailed pseudoanonymous records of their illness using structured and quantitative tracking tools. However, PLM has limitations because, currently, all data are patient-reported and no validating information is required. Thus, some members may not be formally diagnosed with the conditions they report and some could intentionally (or unintentionally) enter erroneous data. The quality of self-reported data may be related to patients’ level of technological comfort, cognitive abilities, degree of motivation, and willingness to return to the site to enter data longitudinally. Websites dependent on voluntary self-reporting suffer from significant attrition over time [9]. As a newer form of data in the health care ecosystem, there are additional steps to be performed such as comparing data quality to traditional data and checking for external validity that might affect generalizability of findings [8].

## Objectives

In this pilot study, we investigated the feasibility of linking a small sample of patients from 2 large data sources at the patient level: the IMS Health database of medical and retail pharmacy claims, covering >250 million lives in the United States, and PLM, a PPRN hosting over 325,000 patients with chronic life-changing illnesses [6]. A secondary objective was to describe the concordance of some common data elements between the 2 datasets.

## Methods

### Data Sources

Members of PLM are prompted to voluntarily report information on their condition history (eg, first symptom date and diagnosis date), relevant symptoms, treatments taken, and laboratory results, and broader health metrics such as weight and quality of life. For the purposes of this pilot study, diagnosis status and treatments were selected as the entities most suitable for matching with the IMS Health database.

Medical and pharmacy billing data are available for purchase for the purposes of research. For this study, an extract of medical and pharmacy (preadjudicated) claims from the IMS Integrated Data Warehouse with dates of service from December 2009 to December 2014 was used. Claims were from office-based physicians and specialists (noncash visits only). Pharmacy claims included prescription data collected from retail, long-term care, specialty, and mail order computerized pharmacy records (both insured and cash transactions). Although the Integrated Data Warehouse data source is not a closed system (ie, not all records from all patients are captured), coverage is wide, representing more than 1 billion medical service records annually as well as an estimated 75% of all prescriptions dispensed in the United States.

### Patients

Multiple sclerosis (MS) was the condition of primary interest in this study, PD patients were included as non-MS controls as a comparator to evaluate the accuracy of MS diagnosis reporting. From the range of common conditions on the PLM system at the time of study, both conditions had relatively large engaged communities, were diagnosed by specialists, have relatively low misdiagnosis rates, and can be identified by the presence of DMTs that are quite specific to each condition. Based on prior studies conducted by PLM, and the pilot nature of this study, target enrollment for this study was 350 actively engaged patients from the PLM communities for MS and PD (approximately 4:1 ratio based on prevalence of the reported diagnoses in the PLM database).

The recruiting process occurred over a 2-week period in early December 2014 and included PPRN patients who met the following eligibility criteria: (1) reported MS or PD on their profile; (2) “actively engaged,” defined as having logged into the PPRN at least once in the 90 days before December 8, 2014; (3) aged 18 years or older at the time of study; (4) account was maintained by the patient; and (5) resided in the United States in the last 10 years.

Eligible patients were sent an invitation on December 8, 2014 to participate in the study via email and private message. Patients who reviewed the message within the 2-week enrollment period could click on a link to a research subject information page where a written statement of research information with informed consent was presented. The research protocol was approved by the New England Institutional Review Board on December 3, 2014. Eligible consenting participants were asked to provide their name, previous last names, date of birth, and zip codes for the last 10 years. This personal health information provided during consent was entered into third-party software to generate encrypted, deidentified (De-ID) tokens. The tokens were then used to make a deterministic match to similarly anonymized patients with claims in the IMS Health database. No payments were made to patients either for taking part in this study or for joining PLM.

**Figure 1:**
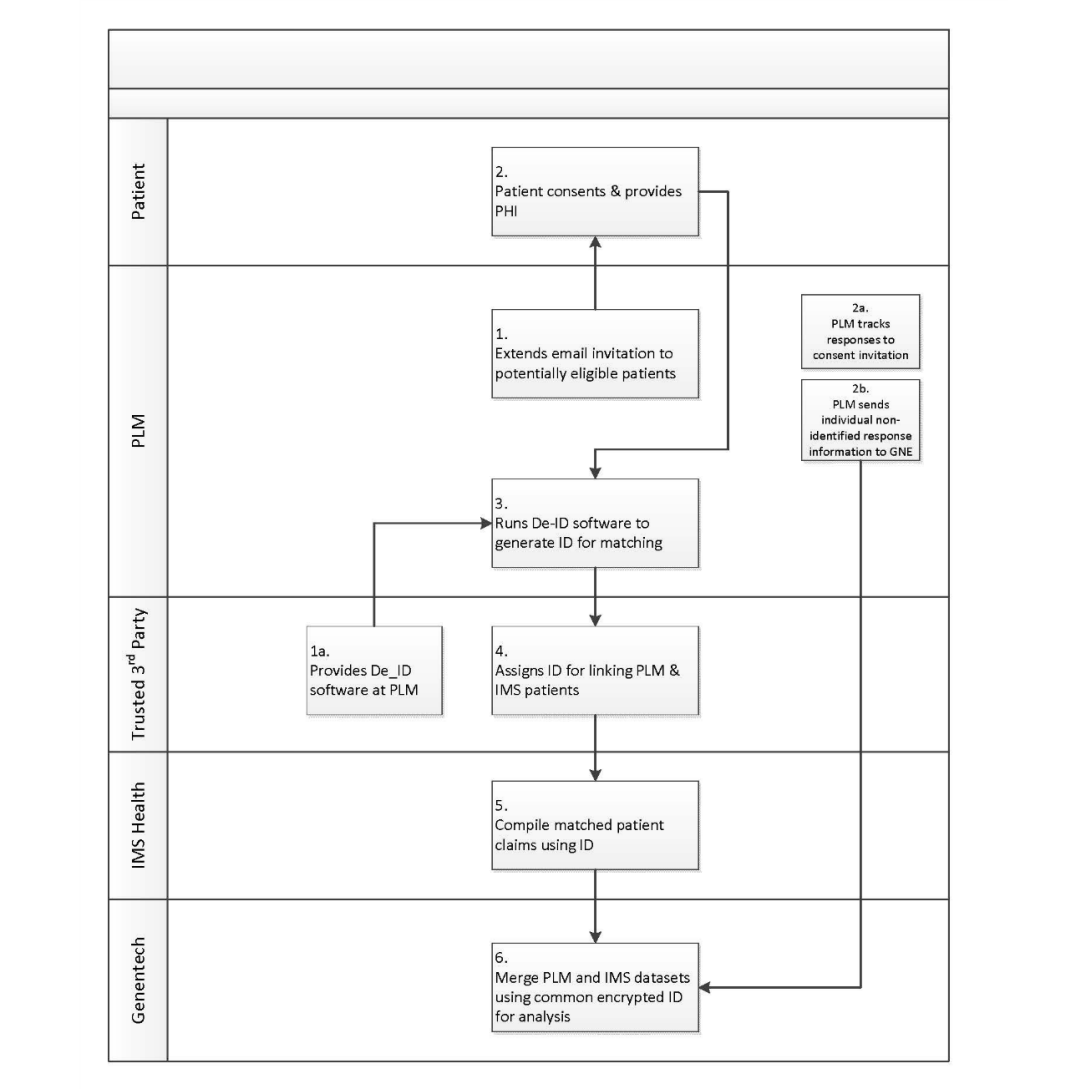
Overview of record matching process.

No data were exchanged between the 2 dataset owners, and all personal health information remained at each source. Data from consenting patients were sent directly to Genentech (GNE) for analysis using encrypted IDs, generated from third party software, for the match. A detailed review of the linking methodology is beyond the scope of this paper. However, extensive research has previously been conducted using this methodology [10], and Figure 1 provides an overview of the matching process used here.

### Analysis Methods

Detailed patient disposition summaries were completed. Descriptive statistics of demographic characteristics were calculated for invited eligible patients, consenting patients (with or without a match), and nonconsenting patients. Additional evaluations of PPRN profile data were explored for patients without matching identity, diagnosis, or drug information in the claims dataset.

The primary objective of the analysis was to explore the feasibility of patient-level linking in the 2 datasets. The overall patient match rate between the 2 sources was calculated as the percentage of consenting patients from PLM with a matching record (De-ID) in the claims database. The secondary objectives were to further explore the concordance between the data sources at the diagnosis and, for MS patients, treatment levels.

**Figure 2:**
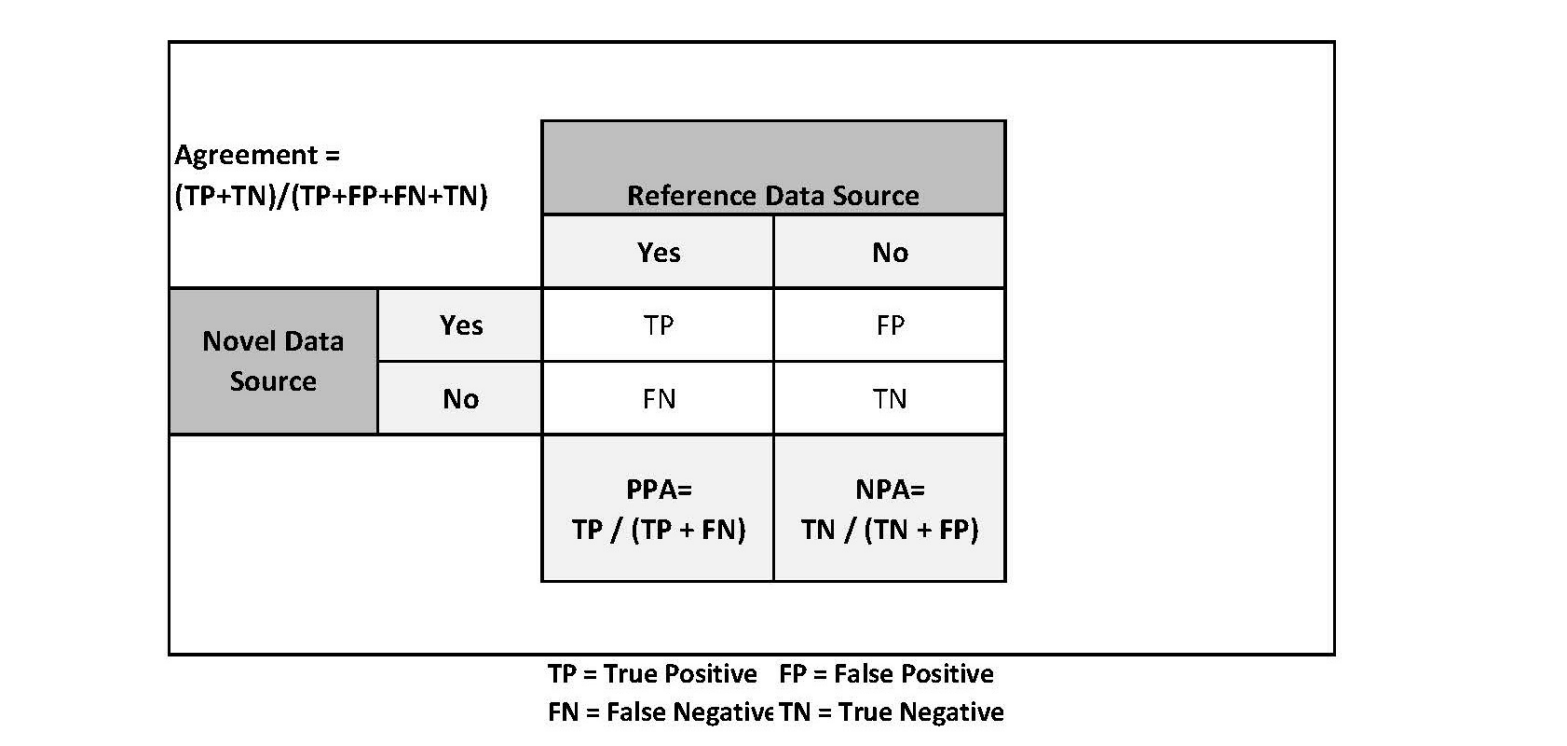
Calculation of agreement.

Two-by-two tables were constructed to evaluate the concordance of diagnosis (MS or not MS) and DMT use (none vs any), and overall (raw) agreement was calculated. Positive percent agreement (PPA) and negative percent agreement (NPA) were calculated, using IMS claims data as the reference (Figure 2). Because the extracted claims dataset may not include all the claims that patients have and treatment reporting in PLM is completely optional, neither data source can truly be considered the “gold standard” and so some discordance between the 2 data sources was expected.

For the evaluation of diagnoses (MS vs not MS), a patient was considered to have MS in the PLM dataset if they had reported MS as a condition in their profile. In the event that a patient reported both MS and PD in their profile, the patient was classified as MS. A patient was considered to have MS in the IMS dataset if they either had at least one medical claim with the International Classification of Diseases, 9^th^ Revision (ICD9) diagnosis code for MS (“340”) or they had a pharmacy claim for one of the following known MS medications, brand name (chemical name, manufacturer): Ampyra (dalfampridine, Acorda), Aubagio (teriflunomide, Genzyme), Avonex (interferon beta 1-a, Biogen), Betaseron (interferon beta 1-b, Bayer), Copaxone (glatiramer acetate, Teva), Extavia (interferon beta-1b, Novartis), Gilenya (fingolimod, Novartis), Lemtrada (alemtuzumab, Genzyme), Novantrone (mitoxantrone, EMD-Serono), Rebif (interferon beta 1-a, EMD-Serono & Pfizer), Tecfidera (dimethyl fumarate, Biogen), Tysabri (natalizumab, Biogen), and Plegridy (peginterferon beta-1a, Biogen).

For the evaluation of MS treatments, the analysis population was restricted to patients with MS as identified in their PLM profile. Analyses were completed for all data (any DMT vs no DMT) and for specific DMTs (yes or no). DMTs include all those drugs listed previously except Ampyra (dalfampridine), which is indicated for the improvement of walking in MS. Spelling variations for DMTs in both datasets were manually adjudicated to ensure appropriate matching. Additional analyses were completed limiting treatment data to those patients reporting treatment within the last 5 years, corresponding to the claims data available for this project. Because many patients did not report start or stop dates for their medications, some analyses were also completed with missing dates imputed as the system-generated dates on which the patients entered specific treatments in their profiles. This imputation permitted additional patient treatment records to fall within the last 5 years.

All analyses were completed using SAS software, version 9.2 for PC (Copyright © 2002-2008, SAS Institute Inc.). SAS and all other SAS Institute Inc. product or service names are registered trademarks or trademarks of SAS Institute Inc., Cary, NC, USA.

## Results

Of the 5364 patients invited via private message through the PPRN website, 2039 (38.0%) viewed the initial invitation, 3325 (61.9%) patients did not open the invitation and, therefore, did not actively accept or decline to participate in the study. Of the 2039 patients who viewed the study invitation, 1421 patients failed to complete the questionnaire, 15 patients actively declined to consent to take part in the study, and, ultimately, 603 patients consented to the study and completed the questionnaire (30% participation rate [603 of 2039] and 11% response rate [603 of 5364]). The target recruitment goal was reached in 24 hours, though the questionnaire remained available for 15 days. Among the 603 consenting patients, 414 reported having MS (69%), 188 reported having PD (31%), and 1 reported having both MS and PD.

### Patient-Level Match

Overall, 567 of the 603 consenting patients (94%) were found in the claims dataset (based on a record with a corresponding De-ID token) and were, therefore, revealed to be real patients with confirmed identities for the purposes of this study. A total of 36 patients were not found within the claims dataset. Two patients subsequently asked to be excluded from the analysis and the PLM community, so the final linked dataset contained 565 matched patients.

### Demographics

Demographic characteristics from PLM profiles for those patients who were invited, consented, matched, and did not match can be found in Table 1. Patients who consented had higher rates of PD, diagnosis by physician (for either condition), reported DMT use, and nonmissing insurance type than nonconsenting patients. All of these factors, except PD diagnosis, would be expected to be related to likelihood of match because diagnosis and treatment by a physician would increase the likelihood of medical and/or pharmaceutical services within claims data.

### Concordance of Diagnosis

The patient-reported MS diagnosis status was confirmed in the claims dataset for 524 (92.7%) matched patients (Table 2). PPA—having MS diagnosis in PLM when MS diagnosis exists in IMS Health—was 99.7%; whereas NPA—not having MS reported in PLM when no MS claim exists in IMS—was 81.1%.

Further analyses were undertaken to better understand potential reasons for mismatch on diagnosis. The 41 patients with discordant MS status in the 2 data sources tended to have fewer medical and pharmacy claims, mean (standard deviation) values are 16.6 (20.74) and 92.9 (242.10), respectively, than patients with concordant MS status, mean (standard deviation) values are 43.1 (44.40) and 206.9 (236.70), respectively. Although the sample sizes were small, patients with concordant MS status were more likely to have a physician diagnosis (96%) than patients with discordant status (88%). Discordant and concordant patients tended to have similar diagnosis date distributions (ie, duration of illness) as well as similar types of insurance (eg, missing, private employer or union based, government funded).

**Table 1:** Demographic characteristics.

Patient Characteristics^a^		Patients invited^b^ (N=5,362)	Patients who did not consent (N=4,759)	Consenting patients with claims^b^ match (N=565)	Consenting patients with no claims match (N=36)
Age in yrs (SD)		54.7 (11.53)	54.3 (11.60)	57.4 (10.63)	56.5 (11.02)
Number of Females (%)		3,546 (66.1)	3,136 (65.9)	385 (68.1)	25 (69.4)
**Primary condition in PLM**					
	MS (%)	3,869 (72.2)	3,470 (72.9)	379 (67.1)	20 (55.6)
	PD (%)	1,333 (24.9)	1,151 (24.2)	168 (29.7)	14 (38.9)
	Other (%)	160 (2.9)	138 (2.9)	18 (3.2)	2 (5.6)
Patient Reports MS or PD Diagnosed by Physician (%)		4,512 (84.2)	3,934 (73.4)	544 (96.3)	34 (94.4)
PLM patients with MS as primary or secondary condition (%)		3,976 (74.2)	3,564 (74.9)	392 (69.4)	21 (58.3)
**MS subtype (% MS)**					
	Relapsing-Remitting	2,429 (61.1)	2,165 (60.8)	250 (63.8)	14 (66.7)
	Primary progressive	253 (6.4)	227 (6.4)	25 (6.4)	1 (4.76)
	Secondary progressive	551 (13.9)	470 (13.2)	78 (19.9)	3 (14.3)
	Progressive relapsing	127 (3.2)	116 (3.3)	10 (2.6)	1 (4.8)
	Unreported	616 (15.5)	586 (16.5)	29 (7.4)	2 (9.5)
**Years since MS Diagnosis^a^ (%)**					
	0 - ≤5 Years	581 (14.6)	524 (14.7)	53 (13.5)	4 (19.05)
	>5 - ≤10 Years	1,102 (27.7)	980 (27.5)	116 (29.6)	6 (28.6)
	>10 - ≤15 Years	711 (17.9)	619 (17.4)	87 (22.2)	5 (23.8)
	>15 - ≤20 Years	408 (10.3)	358 (10.0)	48 (12.2)	2 (9.5)
	>20 Years	566 (14.3)	492 (10.8)	71 (18.1)	3 (14.3)
	[Not Reported]	609 (15.3)	591 (16.6)	17 (4.3)	1 (4.8)
**Reported MS DMT use in PLM (%)**		3,118 (78.4)	2,751 (77.2)	351 (90.0)	16 (76.2)
**Reported Insurance Type (%)**					
	Indian Health Service	1 (0.02)	1 (0.02)	0 (0.0)	0 (0.0)
	Medicaid/ other low-income plan	195 (3.64)	165 (3.47)	29 (5.13)	1 (2.78)
	Medicare	1023 (19.08)	799 (16.79)	209 (36.99)	15 (41.67)
	National health service	7 (0.13)	7 (0.15)		
	Other type of insurance	49 (0.91)	42 (0.88)	6 (1.06)	1 (2.78)
	Private (individual plan)	210 (3.92)	183 (3.85)	27 (4.78)	
	Private (via employer /union)	1351 (25.20)	1141 (23.98)	203 (35.93)	7 (19.44)
	TRICARE (or oth military ins)	55 (1.03)	47 (0.99)	7 (1.24)	1 (2.78)
	Veteran's Administration	69 (1.29)	54 (1.13)	13 (2.30)	2 (5.56)
	No Insurance	87 (1.62)	79 (1.66)	6 (1.06)	2 (5.56)
	Prefer not to answer	77 (1.44)	74 (1.55)	2 (0.35)	1 (2.78)
	[Not Reported]	2238 (41.74)	2167 (45.53)	63 (11.15)	6 (16.67)

^a^Source for all characteristics is PLM; all statistics reported are n (%) unless otherwise noted.

^b^Two patients who were invited, consented and had at least 1 claim in the claims dataset asked to have their profiles removed from PLM and are, therefore, not represented in this analysis.

**Table 2:** Concordance of data sources on diagnosis.

MS reported by patient^a^	MS claim^b^			Agreement^c^		
	MS	Not MS	Total	Overall	PPA	NPA
MS	352	40	392	92.7%	99.7%	81.1%
Not MS	1	172	173			
Total	353	212	565			

^a^MS reported by patient means that patient did or did not report MS in their patient profile.

^b^MS claim means that patient did or did not have a claim with a diagnosis code for MS or a claim for a drug uniquely indicated for treatment of MS.

^c^PPA and NPA calculations use claims datasource as reference.

### Concordance of DMT Use for Patients Reporting MS

Patients reporting MS on their profile had an overall agreement of 58.7% for DMT usage between the datasets, with high PPA (97%) and low NPA (18.3%; top section of Table 3). To explore the possibility that low overall agreement was due to higher rates of DMT use more than 5 years ago (before claims extract for this analysis), patient-reported DMT use was categorized based on use within the past 5 years only. For the purposes of determining patient-reported medication use within 5 years, missing DMT use dates were treated as no DMT within 5 years and, separately, imputed using date of medication data entry by patient (second and third sections of Table 3). A shift of patients to “no DMT use within 5 years” in the PPRN source was observed, but overall agreement between data sources remained similar. When missing dates were imputed, the number of patients using DMTs within 5 years increased from 188 to 284, PPA was increased by 25.4%, and NPA was decreased by 23.6%.

Analyses of specific DMT usage based on 5-year categories with imputation of missing dates revealed an overall agreement ranging from 73.5% to 100% depending on the DMT. PPA and NPA were >50% for most DMTs. Complete results are provided in Table 4.

**Table 3:** Concordance of data sources on any DMT use in MS patients.^a^

Imputation	Patient-reported MS medication	MS medication in claims (5 years)			Agreement^b^		
		Any DMT	No DMT	Total	Overall	PPA	NPA
	Overall any DMT	195	156	351	58.7%	97.0%	18.3%
No	Overall no DMT	6	35	41			
	Total	201	191	392			
No	Any DMT (5 years)	111	77	188	57.4%	55.2%	59.7%
	No DMT (5 years)	90	114	204			
	Total	201	191	392			
Yes	Any DMT (5 years)	162	122	284	58.9%	80.6%	36.1%
	No DMT (5 years)	39	69	108			
	Total	201	191	392			

^a^DMT use categories in this table reflect claims or patient reported; if no dates available then included under “no” for no imputation; missing dates for DMT use imputed based on date of medication entry to evaluate use within 5 years in section marked “yes.”

^b^PPA and NPA calculations use claims as reference.

**Table 4:** Concordance of data sources on specific DMT use within 5 years in PLM MS patients.^a^

	Patient-reported MS medication	MS medication in claims		Agreement^b^		
		Yes	No	Overall	PPA	NPA
Alemtuzumab	Yes	0	0	100.0%	-	100.0%
	No	0	392			
Dimethyl fumarate	Yes	47	60	83.4%	90.4%	82.4%
	No	5	280			
Fingolimod	Yes	24	27	91.8%	82.8%	92.6%
	No	5	336			
Glatiramer acetate	Yes	49	72	74.0%	62.0%	77.0%
	No	30	241			
Interferon Beta 1a	Yes	37	86	73.5%	67.3%	74.5%
	No	18	251			
Interferon Beta 1b	Yes	11	38	87.2%	47.8%	89.7%
	No	12	331			
Metoxantrone	Yes	0	17	95.7%	-	95.7%
	No	0	375			
Natalizumab	Yes	15	51	84.2%	57.7%	86.1%
	No	11	315			
Peginterferon Beta 1	Yes	0	0	99.7%	0.0%	100.0%
	No	1	391			
Teriflunomide	Yes	3	13	94.9%	30.0%	96.6%
	No	7	369			

^a^DMT use categories in this table reflect claims or patient reported; missing dates for DMT use imputed based on date of medication entry to evaluate use within 5 years.

^b^PPA and NPA calculations use IMS claims as reference.

## Discussion

### Principal Findings

This study demonstrated the feasibility of linking patient-reported data with billing claims data generated in the clinical setting. The study surpassed its expected degree of patient engagement (n=350), having gained consent from 603 patients. This suggests that patients were more open to, and supportive of, this project than first anticipated.

The degree of concordance between the PPRN and claims datasets was high, with 94% agreement on patient identity, 93% agreement on MS diagnosis status (MS vs not MS), and agreement on specific DMT usage ranged from 74% to 100%. Finally, the results indicate that these patients were willing and able to accurately recount their diagnosis and to a lesser degree, their use of DMTs in MS care. In total, these findings support the conclusion that accurate linkages at the patient level are possible, opening the doors for further research on an enriched dataset.

Although the rates of patient matching and concordance of diagnosis were high, a careful review of findings and limitations is important to better understand the context and implications for future research. For example, some discordance or nonoverlap of the 2 data sources should be expected a priori. At the patient level, corresponding record(s) in the data sources would not be expected in the following situations: if patients paid for physician services in cash, were uninsured, insured by payers not reflected in the IMS Health database, or received medications via distributers not reflected in the IMS Health database. Analysis of PLM patient profile data revealed that the 36 patients without any claims had lower rates of private insurance than the 565 patients with claims (19.4% vs 35.9%, respectively). Thus, it is possible that at least some of these patients had care that was not captured in the claims dataset.

At the diagnosis and treatment levels, a match to the claims system would not be expected in several situations such as if a patient received treatment for their condition before December 2009 or too recently (because there is a lag between date of service and billing claim submission) or joined a disease community without having been diagnosed by a physician. Conversely, we might see claims without corresponding information in the PPRN if the patient forgot or chose not to report certain information. Patients reported both current and retrospective diagnoses and treatments—so timing did not appear to be a major driver of match rates. Patients with a match were more likely to report having a physician diagnosis (96%) than patients without a match (88%). A larger percentage of diagnosis-unmatched patients reported having Primary Progressive MS, than those who did have a matching diagnosis (15% vs 4%, respectively). Because there are no approved treatments for this subtype of MS, these patients are less likely to be taking a DMT and are thus less likely to have a treatment claim in the IMS database [11,12]. Finally, patients without matching diagnoses in both datasets tended to have fewer medical and pharmacy claims than patients with a match, thus decreasing the chances of a match on a diagnosis category or specific treatment.

The characteristics of each dataset must also be considered to better understand the potential value of linking them to create an enriched dataset. The PLM community is a self-selected population that is biased somewhat to be more educated, female, white, and technologically savvy [13,14]. PLM collects voluntary reports that may result in data gaps on care received or health status. In addition, the patients who consented into this study may represent a more engaged and activated population than those individuals who did not consent. Although this study does represent the findings of linking the PLM PPRN to a claims dataset, attempting to generalize the findings to other studies of linked data sources without regard to their designs, patient inclusion factors, and response characteristics may be unrealistic.

Medical and prescription claims databases are a widely used tool for exploring how real-world healthcare services and treatments are used by physicians and patients. Claims data has long been a core resource for health economics and outcomes research, therapeutic persistence and utilization studies, quality, and other core areas of health services research [15]. Nonetheless, claims data has potentially significant limitations including the time lag of 6-9 months in complete reporting because of the claims filing and adjudication processes [15,16], the inability to capture clinical outcomes of health care interventions more precisely, and the lack of explanations for why something happened. For example, when an MS patient experiences a relapse in their disease they may present to a hospital, be subjected to multiple tests, be provided new medications, and even switched to another DMT. Although the services and treatments used may be captured within the claims dataset, the initiating event, the relapse, is never explicitly captured in the claims dataset, forcing the health services researchers to infer the relapse event using a complex and likely imperfect algorithm [15,17]. The potential real-time nature of patient-generated and patient-reported data provide an opportunity to capture an entirely new dimension of the patient’s health care experience, that is, their account of what happened, although these are of course subject to self-reporting biases [18].

Although the levels of concordance and generalizability of these results to diseases other than MS has not been demonstrated within this study, the high degree of concordance between the patient-reported and claims-based datasets observed here suggests that it may be possible to use these types of combined datasets to answer new research questions. For example, because the PPRN captures treatment experience data (side effect severity, specific side effects, perceived efficacy, and so forth) one could create treatment persistence curves that are stratified by the patient’s experience. This would enable researchers to get at the “why” behind treatment decision starts and stops. Similarly, given that PLM enables patients to synchronize wearable devices to their profile [19], one could look at how health care utilization patterns may differ based on the physical activity of a patient. In addition, patient preferences might be obtained from questionnaires and then linked to their claims data to quantify differences in health care utilization. These sorts of questions would be difficult or impossible to answer in the absence of a linked dataset.

### Conclusion

Real-world datasets and patient-reported data are becoming increasingly powerful tools for research, quality improvement, and broader understanding of the evolving health care system. The further integration of traditional claims or healthcare-system generated data, coupled with patient-reported data will continue to bring about ever new opportunities to study disease, patient experience, and the applications of health care services and their effects on the health care system and patient outcomes. The generalizability of our findings and patient willingness to consent to data linkage for clinical research needs further exploration in other settings.
