# Correction of: Exploring Concordance of Patient-Reported Information on PatientsLikeMe and Medical Claims Data at the Patient Level

**DOI:** 10.2196/jmir.6757

**Published:** 2016-10-27

**Authors:** Gabriel S Eichler, Elisenda Cochin, Jian Han, Sylvia Hu, Timothy E Vaughan, Paul Wicks, Charles Barr, Jenny Devenport

**Affiliations:** ^1^PatientsLikeMeCambridge, MAUnited States; ^2^GenentechMedical AffairsSouth San Francisco, CAUnited States

The authors of, “Exploring Concordance of Patient-Reported Information on PatientsLikeMe and Medical Claims Data at the Patient Level” (J Med Internet Res 2016;18(5):e110) would like to make changes in the fourth paragraph under the heading of Principal Findings in the Discussion section of the paper.  

The text should read, “A larger percentage of diagnosis-unmatched patients reported having Primary Progressive MS, than those who did have a matching diagnosis (15% vs 4%, respectively).” instead of “A larger percentage of diagnosis-unmatched patients reported having primary progressive MS than those who did have a matching diagnosis (15% vs 3%, respectively).”

The second correction is in Table 1. There were missing cells that lead to the absence of a key MS subgroup, relapsing-remitting MS, with knock-on consequences for other cells in that table. 

1. The value of “Age in years (SD)” was changed in the column “Consenting patients with claims match” from 47.4 (10.63) to 57.4 (10.63).

2. The value of “PLM patients with MS as primary or secondary condition (%)” was changed in the column “Consenting patients with claims match” from 391 (69.2) to 392 (69.4). 

3. The row “Relapsing-remitting” was added under “MS subtype.” The values, 2,429 (61.1), 2,165 (60.8), 250 (63.8), and 14 (66.7) were added for columns “Patients invited (N=5,362),” “Patients who did not consent (N=4,759),” “Consenting patients with claims match (N=565),” and “Consenting patients with no claims match (N=36),” respectively. 

4. The values for the row “Primary progressive” under “MS subtype” were changed from 2682 (67.5), 2392 (65.5), 275 (70.3), and 15 (71.4) to 253 (6.4), 227 (6.4), 25 (6.4), and 1 (4.76) in each column, respectively.

5. The values for the row “Unreported” under the “MS subtype” were changed from 676 (18.5) and 28 (7.2) to 586 (16.5) and 29 (7.4) under columns “Patients who did not consent (N=4,759)” and “Consenting patients with claims match (N=565),” respectively.

6. The percentages have also been adjusted in the “Reported MS DMT use in PML” category from 3118 (58.2), 2751 (57.8), 351 (62.1), and 16 (44.4) to 3,118 (78.4), 2,751 (77.2), 351 (90.0), and 16 (76.2) in each column, respectively.

The corrected Table 1 is as below:

**Table 1:** Demographic characteristics.

Patient Characteristics^a^		Patients invited^b^ (N=5,362)	Patients who did not consent (N=4,759)	Consenting patients with claims^b^ match (N=565)	Consenting patients with no claims match (N=36)
Age in yrs (SD)		54.7 (11.53)	54.3 (11.60)	57.4 (10.63)	56.5 (11.02)
Number of Females (%)		3,546 (66.1)	3,136 (65.9)	385 (68.1)	25 (69.4)
**Primary condition in PLM**					
	MS (%)	3,869 (72.2)	3,470 (72.9)	379 (67.1)	20 (55.6)
	PD (%)	1,333 (24.9)	1,151 (24.2)	168 (29.7)	14 (38.9)
	Other (%)	160 (2.9)	138 (2.9)	18 (3.2)	2 (5.6)
Patient Reports MS or PD Diagnosed by Physician (%)		4,512 (84.2)	3,934 (73.4)	544 (96.3)	34 (94.4)
PLM patients with MS as primary or secondary condition (%)		3,976 (74.2)	3,564 (74.9)	392 (69.4)	21 (58.3)
**MS subtype (% MS)**					
	Relapsing-Remitting	2,429 (61.1)	2,165 (60.8)	250 (63.8)	14 (66.7)
	Primary progressive	253 (6.4)	227 (6.4)	25 (6.4)	1 (4.76)
	Secondary progressive	551 (13.9)	470 (13.2)	78 (19.9)	3 (14.3)
	Progressive relapsing	127 (3.2)	116 (3.3)	10 (2.6)	1 (4.8)
	Unreported	616 (15.5)	586 (16.5)	29 (7.4)	2 (9.5)
**Years since MS Diagnosis^a^ (%)**					
	0 - ≤5 Years	581 (14.6)	524 (14.7)	53 (13.5)	4 (19.05)
	>5 - ≤10 Years	1,102 (27.7)	980 (27.5)	116 (29.6)	6 (28.6)
	>10 - ≤15 Years	711 (17.9)	619 (17.4)	87 (22.2)	5 (23.8)
	>15 - ≤20 Years	408 (10.3)	358 (10.0)	48 (12.2)	2 (9.5)
	>20 Years	566 (14.3)	492 (10.8)	71 (18.1)	3 (14.3)
	[Not Reported]	609 (15.3)	591 (16.6)	17 (4.3)	1 (4.8)
**Reported MS DMT use in PLM (%)**		3,118 (78.4)	2,751 (77.2)	351 (90.0)	16 (76.2)
**Reported Insurance Type (%)**					
	Indian Health Service	1 (0.02)	1 (0.02)	0 (0.0)	0 (0.0)
	Medicaid/ other low-income plan	195 (3.64)	165 (3.47)	29 (5.13)	1 (2.78)
	Medicare	1023 (19.08)	799 (16.79)	209 (36.99)	15 (41.67)
	National health service	7 (0.13)	7 (0.15)		
	Other type of insurance	49 (0.91)	42 (0.88)	6 (1.06)	1 (2.78)
	Private (individual plan)	210 (3.92)	183 (3.85)	27 (4.78)	
	Private (via employer /union)	1351 (25.20)	1141 (23.98)	203 (35.93)	7 (19.44)
	TRICARE (or oth military ins)	55 (1.03)	47 (0.99)	7 (1.24)	1 (2.78)
	Veteran's Administration	69 (1.29)	54 (1.13)	13 (2.30)	2 (5.56)
	No Insurance	87 (1.62)	79 (1.66)	6 (1.06)	2 (5.56)
	Prefer not to answer	77 (1.44)	74 (1.55)	2 (0.35)	1 (2.78)
	[Not Reported]	2238 (41.74)	2167 (45.53)	63 (11.15)	6 (16.67)

^a^Source for all characteristics is PLM; all statistics reported are n (%) unless otherwise noted.

^b^Two patients who were invited, consented and had at least 1 claim in the claims dataset asked to have their profiles removed from PLM and are, therefore, not represented in this analysis.

Both these alterations have been made in the online version of the paper on the JMIR website on October 27, 2016 together with publishing this correction notice. Because these were made after submission to PubMed and other full-text repositories, the correction notice has been submitted to PubMed, and the original paper has been resubmitted to PubMed Central. The corrected metadata have also been resubmitted to CrossRef.

